# Optimizing Clinical Decision Support for Antibiotic Prescribing in Pediatric Acute Respiratory Tract Infections: A Usability Study

**DOI:** 10.1055/a-2906-3186

**Published:** 2026-07-22

**Authors:** Janet Lee, Dina Abdel-Rahman, Emma Kelly, Praveena Korakuti, Erin Tagai

**Affiliations:** 1 12314 Lewis Katz School of Medicine, Temple UniversityPhiladelphiaPennsylvaniaUnited States; 2 Department of Cancer Prevention and Control 6565 Fox Chase Cancer CenterPhiladelphiaPennsylvaniaUnited States

**Keywords:** clinical decision support, usability testing, implementation science, antimicrobial stewardship

## Abstract

**Background:**

Inappropriate antibiotic prescribing contributes to antimicrobial resistance. Antimicrobial stewardship (AMS) programs increasingly rely on electronic health record (EHR)-based clinical decision support (CDS) alerts to guide evidence-based care, yet poorly designed alerts can increase cognitive burden and contribute to burnout. Usability testing offers a structured approach to improving alert design and adoption.

**Objectives:**

This study aimed to evaluate and iteratively refine interruptive CDS alerts developed to support appropriate antibiotic prescribing for pediatric acute respiratory tract infections (ARTIs) within an urban academic health system as part of a larger implementation project.

**Methods:**

Twelve providers participated in four waves of virtual usability testing using scripted clinical scenarios reflecting common workflows. Participants interacted with three clinical alerts and one design-focused scenario. Quantitative outcomes included task effectiveness, efficiency, satisfaction, and perceived difficulty. Qualitative feedback was gathered via think-aloud techniques and synthesized using a “rainbow spreadsheet.” Iterative modifications were implemented between waves until thematic saturation was achieved.

**Results:**

Task completion across scenarios was high (83.3%). Effectiveness and efficiency improved over waves, and satisfaction increased when alerts embedded actionable tasks such as ordering tests, adding diagnoses, or switching antibiotics. Participants emphasized the importance of clear, concise text and intuitive defaults. Including links to evidence-based guidelines improved trust. Alert refinements reduced cognitive load and improved workflow alignment.

**Conclusion:**

Rapid, iterative usability testing substantially improved the clarity and workflow integration of interruptive CDS alerts. Incorporating usability evaluation into CDS stewardship efforts can enhance adoption, reduce alert fatigue, and strengthen implementation of evidence-based practices.

## Background and Significance

Inappropriate use of antibiotics has been associated with adverse effects on health, most notably in contributing to antibiotic resistance. 1 2 3 Inappropriate use of antibiotics has also been associated with increased rates of *Clostridium difficile* infections, which are associated with significant morbidity and mortality. 1 3 4

Antimicrobial stewardship (AMS) programs have been developed to combat inappropriate antibiotic use. 1 These programs have reduced inappropriate prescribing and positively affected patient outcomes. 1 Interruptive electronic health record (EHR) alerts have been among the most implemented strategies to influence antibiotic prescribing. 2 Clinical decision support (CDS) tools, including interruptive alerts, are effective tools to incorporate evidence-based guidelines into clinical practice. 5 Despite the potential of CDS tools, poorly designed alerts can contribute to burnout 6 7 8 and alert fatigue. 9 CDS stewardship practices, including institutional governance around interruptive alerts, can improve providers' attitudes toward EHR tools. 10

Effective use of CDS tools can positively affect the implementation of new evidence-based guidelines. 11 Governing principles and development teams who design and implement, respectively, should utilize effective implementation strategies when creating CDS systems, including aligning CDS systems with current practice and complex workflows; ensuring front-end usability by developing simple, intuitive, and attractive systems; and engaging stakeholders early and throughout the development process. 11

## Objectives

As part of an urban health system-wide implementation science (IS) project designed to improve pediatric AMS across diverse practice settings for acute respiratory tract infections (ARTIs)—defined as acute otitis media, acute sinusitis, pneumonia, or strep pharyngitis—we used multiple strategies to improve prescribing, including custom CDS tools, care team education, site meetings, and audit-and-feedback reports. Within this broader effort, we developed interruptive EHR alerts to support the implementation of new evidence-based antibiotic prescribing guidelines. 12 We also identified a documentation gap in our prescribing workflow, as antibiotic orders in our health system do not require entry of an indication or reason for prescribing. Practice settings included outpatient pediatrics, family medicine, and general internal medicine clinics, as well as urgent care centers and emergency departments. To promote consistent, efficient implementation across these varied clinical environments and workflows, we deployed interruptive alerts termed “Our Practice Alerts” within the EHR. 13

The alerts were designed and built by two physician builders, practicing clinicians who completed specialized training to configure and customize the Epic EHR system. 13 The objectives of this study were to evaluate the usability of these interruptive alerts and iteratively refine them to improve clarity, workflow integration, and stakeholder acceptance prior to broader implementation. We hypothesized that conducting usability testing with primary care providers would generate significant feedback to create iterative improvements to the approved alerts to decrease alert overrides and improve attitudes toward the interruptive alerts, with the goal of ultimately improving AMS in the larger project. Additionally, we hypothesized that usability testing would be an effective way to engage stakeholders in the larger implementation project.

## Methods

### Study Participants, Recruitment, and Setting

We recruited 12 pediatric and family medicine clinicians—including physicians, nurse practitioners (NPs), and physician assistants (PAs)—who provide care to pediatric patients (aged 0–18 years) in ambulatory practices within an urban academic health system. Ambulatory sites were prioritized because they serve a higher volume of pediatric patients. Recruitment was conducted by email. To capture a range of user perspectives, participants with varying levels of EHR proficiency were enrolled and assigned to four waves of three participants each. Participants were assigned to waves sequentially in the order in which they were recruited. Because participants completed a single usability session and were exposed to only one alert iteration, a washout period was not applicable. Enrollment continued until thematic saturation was achieved.

This project was deemed non-human subjects research by the Temple University Institutional Review Board.

### Study Design

#### Initial Development of Electronic Health Record Alerts

As part of a broader IS project to improve AMS among pediatric patients, three interruptive CDS tools were developed in the Epic EHR testing environment, 13 reproduced with permission from 2026 Epic Systems Corporation. Two physician builders designed the alerts to support documentation and prescribing workflows for pediatric ARTIs based on a Patient-Centered Outcomes Research Institute (PCORI)-funded comparative effectiveness study. 12 The tools developed for this project developed during the preimplementation stage of our IS project were approved for deployment by the institution's information technology (IT) governance committee.

#### Usability Testing Scenarios and Sessions

Based on the alerts, three clinical scenarios and one non-clinical scenario were developed to guide the usability testing sessions. Scenarios were informed by input from pediatric providers and pilot-tested by the physician builders.

**Fig. 1:**
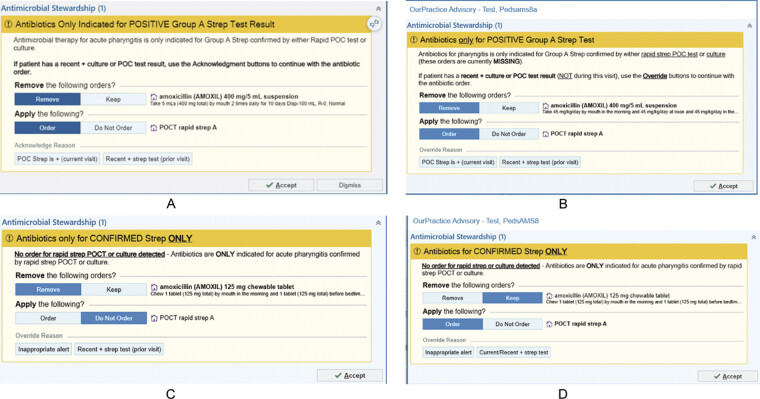
( **A** ) Baseline Group A streptococcal pharyngitis alert. Reproduced with permission from a screenshot of the alert from Epic (source: ©2026 Epic Systems Corporation) on January 23, 2025. ( **B** ) Baseline Group A Streptococcal (GAS) pharyngitis alert (wave 2). Reproduced with permission from a screenshot of the alert from Epic (source: ©2026 Epic Systems Corporation) on January 23, 2025. ( **C** ) Baseline Group A Streptococcal (GAS) pharyngitis alert (wave 3). Reproduced with permission from a screenshot of the alert from Epic (source: ©2026 Epic Systems Corporation) on January 23, 2025. ( **D** ) Baseline Group A Streptococcal (GAS) pharyngitis alert (wave 4). Reproduced with permission from a screenshot of the alert from Epic (source: ©2026 Epic Systems Corporation) on January 23, 2025. POC, point of care.

**Fig. 2:**
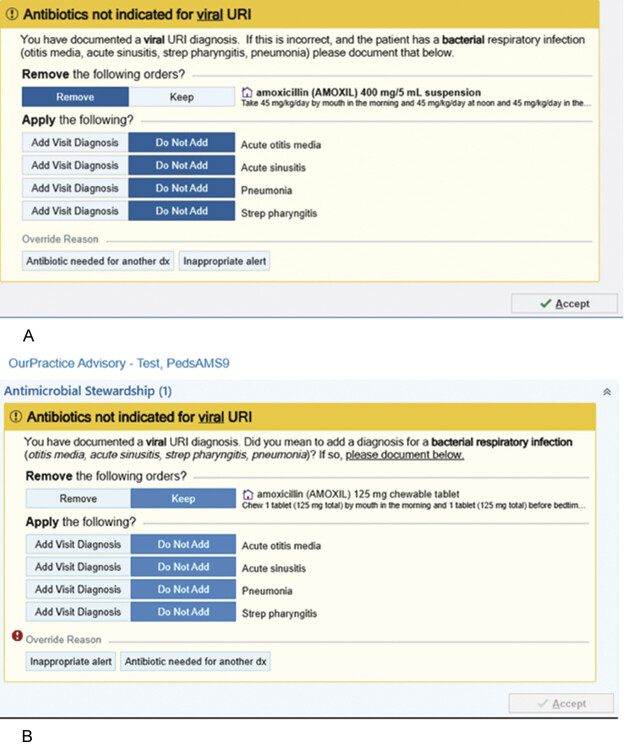
( **A** ) Viral upper respiratory infections alert. Reproduced with permission from a screenshot of the alert from Epic (source: ©2026 Epic Systems Corporation) on January 23, 2025 (baseline). ( **B** ) Viral upper respiratory infections (URIs) alert (wave 4). Reproduced with permission from a screenshot of the alert from Epic (source: ©2026 Epic Systems Corporation) on January 23, 2025.

**Fig. 3:**
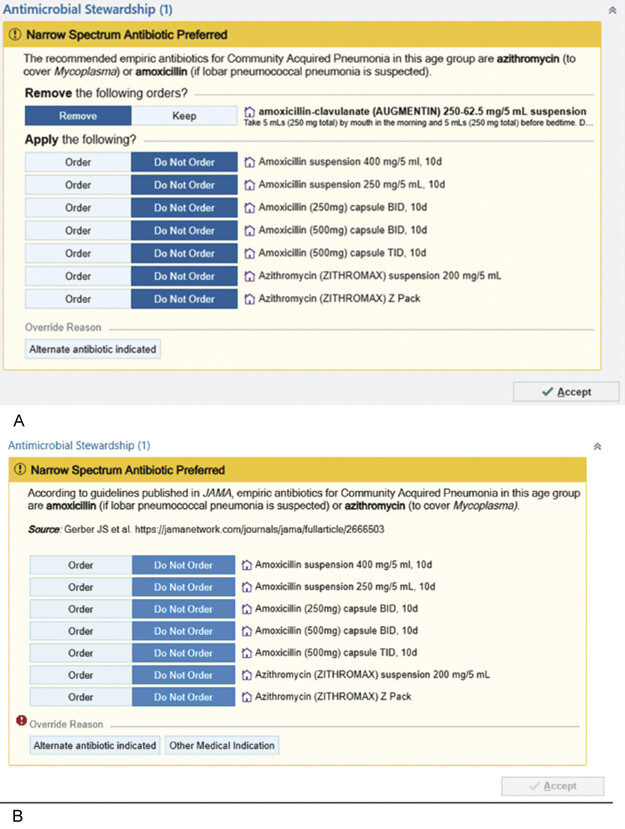
( **A** ) Narrow-spectrum antibiotics (ARTIs) alert. Reproduced with permission from a screenshot of the alert from Epic (source: ©2026 Epic Systems Corporation) on January 23, 2025 (baseline). ( **B** ) Narrow-spectrum antibiotics (ARTIs) alert (wave 4). Reproduced with permission from a screenshot of the alert from Epic (source: ©2026 Epic Systems Corporation) on January 23, 2025. ARTI, acute respiratory tract infection.

**Fig. 4:**
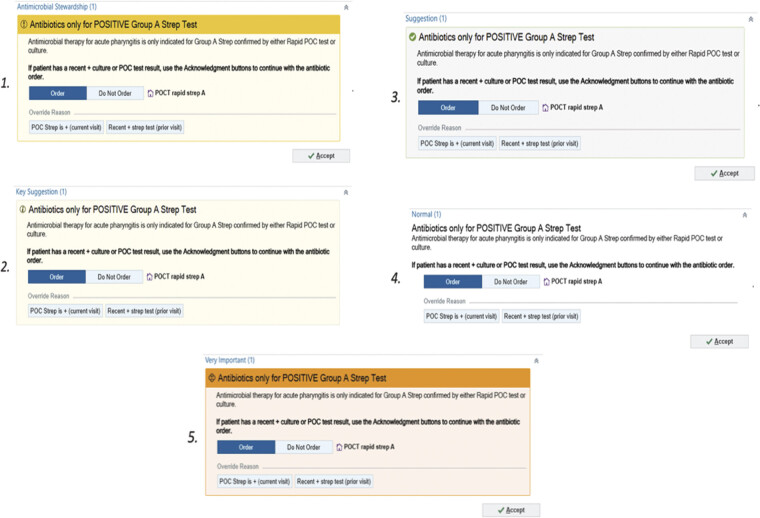
Color scheme choices for alerts. Color scheme choices for alerts: 1. Yellow, 2. Pale Yellow, 3. Gray, 4. White, 5. Orange. Reproduced with permission from a screenshot of the alert from Epic (source: ©2026 Epic Systems Corporation) on January 23, 2025.

1. **Group A streptococcal pharyngitis** : An alert prompting documentation of a Group A streptococcal (GAS) infection prior to prescribing antibiotics ( Fig. 1 ).
  - This alert fires if an antibiotic order is placed for a GAS diagnosis without a positive point-of-care (POC) rapid strep test or culture. The alert also fires when signing the visit if the POC test has not resulted. The alert can be resolved by removing the unsigned antibiotic order or ordering a POC rapid strep test. The alert can also be acknowledged/overridden by marking a positive POC strep in the current visit or a recent positive strep test.
2. **Viral upper respiratory infections** : An alert discouraging antibiotic prescriptions for viral upper respiratory infections (URIs; Fig. 2 ).
  - The alert fires when an order for antibiotics is placed for a diagnosis of viral URI in pediatric patients. The alert can be resolved by removing the unsigned antibiotic order or adding an ARTI diagnosis (typically acute otitis media, acute sinusitis, pneumonia, or strep pharyngitis). The alert can be acknowledged/overridden by marking that the ordered antibiotic is needed for another diagnosis or that this alert is inappropriate for the given clinical situation.
3. **Narrow-spectrum antibiotics** : An alert that discourages broad-spectrum antibiotic prescriptions for ARTIs ( Fig. 3 ).
  - The alert fires if a broad-spectrum antibiotic (e.g., Augmentin) is ordered for an ARTI diagnosis in pediatric patients. Users are presented with narrow-spectrum alternatives (e.g., amoxicillin) and could resolve the alert by removing the unsigned order and selecting an alternative or by overriding the alert to indicate an alternative antibiotic was clinically appropriate.
4. **Visual appearance of alerts** : Comparison of five alert color schemes ( Fig. 4 ).
  - Participants were presented with screenshots of the alert from scenario one in five different color variations.

Each 1-hour virtual session was conducted via Microsoft Teams. 14 Sessions were moderated by the principal investigator of the larger IS project. Participants were given “remote control” access to the moderator's computer to interact with alerts in the EHR testing environment. The moderator guided participants through scripted scenarios reflective of common ambulatory workflows, encouraging participants to use their clinical judgment while verbalizing their thought processes using the think-aloud technique. 15 After each scenario, participants were prompted to reflect on clarity, workflow effect, interruptiveness, design, and emotional response. Additional feedback was encouraged throughout sessions. All participants completed every scenario, and alert refinements were implemented between waves until thematic saturation was achieved.

#### Data Collection: Documentation of Observations

**Fig. 5:**
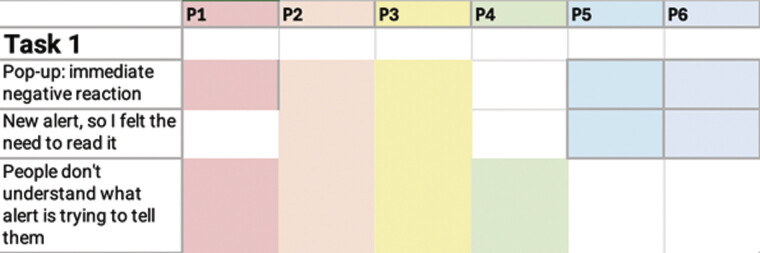
Rainbow spreadsheet.

Two study team members captured observations in real time using a shared “rainbow spreadsheet,” a matrix-based qualitative user design (UX) tool that supports rapid synthesis of feedback across participants and waves. 16 Discrete rows documented observations and qualitative feedback themes, while columns indicated a participant, highlighted by a unique color. Following each session, the three study team members who participated in the usability session conducted debriefs to harmonize observations and themes. After each wave, alerts were iteratively refined until thematic saturation was reached. A portion of the rainbow spreadsheet used by our study team is seen in Fig. 5 .

### Study Measures

#### Usability Testing—Quantitative Measures

During the usability testing sessions, we collected quantitative and qualitative data. Quantitative measures included a binary effectiveness measure, defined as whether tasks were completed correctly. Efficiency was defined as the time for task completion, measured in seconds. Satisfaction was defined as how satisfied participants were with the task, measured on a scale of 1 to 5 (1, very dissatisfied; 5, very satisfied). Difficulty was defined as perceived ease of a task measured on a scale of 1 to 5 (1, very difficult; 5, very easy). Effectiveness and efficiency were measured by the study team, while satisfaction and difficulty were verbally reported by participants.

#### Usability Testing—Qualitative Measures

Qualitative data consisted of participants' verbalized feedback during think-aloud sessions, 15 focusing on usability, workflow integration, emotional response, and trust in clinical guidance. Themes were used to guide iterative alert modifications between waves. 16

#### Survey Data

After usability testing, participants completed an online survey via Research Electronic Data Capture (REDCap) 17 assessing demographics (age, sex, race, ethnicity, position, number of years since terminal training completion, and number of years working at the institution) as well as comfort with new technology, methods for learning new EHR functionality, perceived barriers to adoption, feelings of connection to system-level change, and a net promoter score. Survey data were measured on a Likert scale of 1 to 5. The survey is available in the Supplemental Materials (available in the online version only).

### Statistical Analyses

Descriptive statistics were used to summarize participant characteristics and quantitative usability measures. Continuous variables were reported as means and medians, while categorical variables were reported as frequencies and percentages. All quantitative analyses were conducted in Microsoft Excel and synthesized during team debriefs using a rainbow spreadsheet. 14 16

## Results

### Participant Demographics

Participant demographics are summarized in Table 1 . The sample was evenly distributed across four age groups. Most participants identified as female, half identified as White, and all identified as non-Hispanic. The median time both at the institution and since terminal degree completion was 5 years.

**Table 1:** Total population characteristics ( *N* = 12)

Characteristics	*N* = 12
Age (y)	*n* (%)
25–30	3 (25)
31–35	3 (25)
36–40	3 (25)
41–50	3 (25)
Sex	
Female	11 (91.7)
Male	1 (8.3)
Race	
White	6 (50)
Black or African American	3 (25)
Asian	3 (25)
Alaskan Native	0 (0)
Native Hawaiian or Pacific Islander	0 (0)
Other	0 (0)
Ethnicity	
Hispanic	0 (0)
Non-Hispanic	12 (100)
Other	0 (0)
Position	
Physician (MD/DO)	10 (83.3)
Advanced practice provider (NP/PA)	2 (16.7)
Resident physician (MD/DO)	0 (0)
Other	0 (0)
Department type	
Pediatrics	7 (58.3)
Family and Community Medicine	5 (41.7)
Number of years since terminal training completion	5.0 (3.0, 9.5)
Number of years working at the institution	5.0 (2.5, 7.5)

Abbreviations: NP, nurse practitioner; PA, physician assistant.

### Postparticipation Survey Results

All users (100%, *N* = 12) completed the postsession survey. Most reported feeling very comfortable or somewhat comfortable learning new technology with no or minimal guidance (50% [ *n* = 6] and 50% [ *n* = 6], respectively). Trial-and-error learning and peer assistance were used most often (83.3% [ *n* = 10] and 91.67% [ *n* = 11], respectively), while formal training materials or online resources were less common: Tipsheets (50%, *n* = 6), online tutorials (33.3%, *n* = 4), Epic User Web (16.7%, *n* = 2), or no additional resources (16.7%, *n* = 2). Most frequently cited barriers were time constraints (83.3%, *n* = 10) and lack of training (58.3%, *n* = 7). Half of the participants (50%, *n* = 6) agreed or strongly agreed that involvement in CDS development made them feel more connected to broader system-level change.

### Overall Usability Outcomes across Scenarios

Across all clinical scenarios (Scenarios 1–3), the overall successful task completion rate was 83.3% ( *n* = 10). Perceived difficulty remained low to moderate. Although effectiveness and efficiency improved across waves, satisfaction was highest when alerts embedded key actions that would otherwise require navigation to other areas of the EHR, such as placing an order or adding a diagnosis.

#### Scenario 1—Group A Streptococcal Pharyngitis Alert

The baseline GAS alert is shown in Fig. 2 .

##### Quantitative Outcomes

Most participants (83%, *n* = 10) successfully completed all tasks in scenario 1. With effectiveness improving from 66.7% (wave 1) to 100% in wave 4, efficiency increased as mean time declined from 85.8 seconds (wave 1) to 65.4 seconds (wave 4). Satisfaction increased from 2.3 (wave 1) to 4.0 (wave 4), and perceived difficulty improved across waves (from 3.8 to 4.2).

##### Qualitative Themes and Iterative Changes

Half of the participants (50%, *n* = 6) immediately noted a negative reaction when seeing the interruptive alert. However, most participants (75%, *n* = 9) felt it was important to take the time to read it in detail as it was a new alert. Half of the participants (50%, *n* = 6) found ordering activity buttons within the alert convenient and thought it would improve their workflow.

Iterative changes were made to the interruptive alert based on feedback from participants across four waves. In wave 1, all participants (100%, *n* = 3) reported that the alert guidance was unclear and that subsequent actions were not evident. In response, alert text was sharpened, explicit reasoning was provided, and resolution options were clarified ( Fig. 1B ). However, in wave 2, participants perceived the alert as too wordy, and cognitive load remained high. To address these concerns, the alert text was shortened, formatting was enhanced (bolding and underlining), and the Override Button was revised for clarity. The updated alert is shown in Fig. 1C .

In wave 3, participants found the revised alert easy to understand and reported improved comprehension of alert rationale. Users requested changing the default (e.g., “Keep” the antibiotics and “Order” the POC strep test) to reduce clicks and avoid framing the alert as challenging clinical judgment, instead framing it as a documentation aide within the EHR. An additional edit was made to shorten the title of the alert. The revised alert can be found in Fig. 1D .

In wave 4, participants unanimously reported that the alert was clear, user-friendly, and trustworthy, and indicated it could positively support clinical workflows ( Fig. 1C ).

#### Scenario 2—Viral Upper Respiratory Infections

The baseline viral URI alert is shown in Fig. 2A .

##### Quantitative Outcomes

Most participants (83%, *n* = 10) were able to successfully complete all tasks in scenario 2. Effectiveness improved as usability testing progressed from 66.7% ( *n* = 2) in wave 1 to 100% ( *n* = 3) in wave 4. Efficiency remained stable with no significant changes between waves (wave 1= 58.8 s, wave 2 = 111.8 s, wave 3 = 64.8 s, wave 4 = 65.4 s). Satisfaction with scenario 2 decreased slightly with subsequent waves from 4.1 in wave 1 to 3.7 in wave 4. Perceived difficulty remained unchanged with a mean difficulty score of 3.8 for wave 1 and 3.7 for wave 4. These wave-to-wave differences were descriptive and were not powered for statistical comparison.

##### Qualitative Themes and Iterative Changes

Most participants (91.7%, *n* = 11) highly valued having an activity within the alert to add a diagnosis. Users stated they externally navigate to add a diagnosis and found the activity within the alert would save time, positively affecting their feelings toward the alert. Most participants (91.7%, *n* = 11) felt that the override buttons for the alert were appropriate, and 58.3% ( *n* = 7) of users had positive feelings toward the alert.

Participants in waves 1 and 2 did not provide any feedback that necessitated any modifications to the alert. In wave 3, a participant reiterated sentiments from scenario 1, emphasizing the importance of designing EHR tools that assume correct clinical judgement while identifying potential gaps in documentation. They recommended rephrasing the language to imply a missing diagnosis rather than assuming an incorrect clinical decision. The updated alert is shown in Fig. 2B .

Wave 4 participants expressed positive feelings toward the alert, affirming that the diagnosis buttons nested in the same activity were helpful. No further changes were required.

#### Scenario 3—Narrow-Spectrum Antibiotics

The baseline alert for narrow-spectrum antibiotics is shown in Fig. 3A .

##### Quantitative Outcomes

Overall, 83% of participants ( *n* = 10) were able to complete all tasks in scenario 3 successfully. Effectiveness improved from 66.7% ( *n* = 2) in wave 1 to 100% ( *n* = 3) in wave 4. Efficiency in task completion improved slightly from wave 1 (86.7 s) to wave 4 (83.8 s). Satisfaction with the alert in scenario 3 improved with subsequent waves (3.6 in wave 1 to 4.7 in wave 4). Perceived difficulty remained consistent (4.7 in wave 1 and 4.3 in wave 4). These wave-to-wave differences are descriptive and were not powered for statistical comparison.

##### Qualitative Themes and Iterative Changes

Almost all participants (91.7%, *n* = 11) reported they found the alternate medication options nested within the alert helpful. Users stated that navigating to the ordering activity is time-consuming, while the new alert workflow saved them time. Nearly all participants (91.7%, *n* = 11) noted the alert felt up to date with new evidence-based guidance.

Participants in waves 1 and 2 reported trusting the alert but felt the need to externally verify the new clinical guidance that was presented. Users recommended including links to the clinical recommendations guiding the alert. Although participants reported they might not have time during patient care sessions, it would help to build trust in the alert. A link to the citation for the evidence-based recommendation was included in the alert.

Participants reported finding the alternative medication list nested in the alert helpful. However, “azithromycin” (broad spectrum; inappropriate order) was the first antibiotic on the pick list. This led to confusion among the users as they reported their preference was to choose “amoxicillin” (narrow spectrum; appropriate order), which aligned with evidence-based guidelines, 12 but indicated pressure to choose “azithromycin” because of its location on the list. Updates were made to reorder amoxicillin as the first option.

Participants also requested an additional override button for “Other Medical Indication.” Feedback from waves 1 and 2 were incorporated into the alert and are shown in Fig. 3B .

Participants in waves 3 and 4 indicated that the inclusion of the hyperlinked citation improved their trust in the alert. Users did not feel the need to verify via external sources. Participants indicated that they thought the alert was helpful and did not provide additional feedback for improvements to the tool.

#### Scenario 4—Visual Appearance of Alerts

Participants were presented with screenshots of the alert from scenario 1 in five different color variations ( Fig. 4 ). Participants preferred the standard yellow (33.3%, *n* = 4) color scheme, as it balanced recognizability and appropriateness, and orange (33.3%, *n* = 4) for boosting salience. However, participants reported that an alert associated with the orange color could be perceived as overly urgent.

## Discussion

CDS tools can help implement evidence-based guidelines into practice. 5 Despite this, poorly designed alerts can contribute to practitioner burnout. 6 7 8 The success of usability tools is dependent upon usability factors, including design, navigability, and user satisfaction. Iterative usability testing can systematically address these issues in CDS tools to support effective implementation. 18 19 Like prior studies conducted within primary care settings, we demonstrated that utilizing rapid usability testing methods employing think-aloud practices can be an effective way to make changes to CDS tools and improve provider satisfaction. 15 18 19

Our results reflected similar studies that noted that providers had negative reactions to interruptive alerts. 20 21 Despite this, usability testing participants positively reviewed our unique alert design that incorporated activities (i.e., changing order and entering a diagnosis) that allow providers to address alert concerns within the same activity. Providers believed that including these additional activities would help them to save time in their clinical workflows. Additionally, participants noted that they understood why interruptive alerts were needed but noted that changing the alert to highlight that a user may have forgotten to document something—rather than implying that a provider's clinical decision-making was incorrect—helped improve users' attitudes toward the alerts. The sequence in which antibiotic options were presented also influenced prescribing choices. 22 Finally, providers acknowledged that EHR alerts could help them to improve patient outcomes, but noted that including a citation to the evidence-based guidelines within the alert improved their trust. 23 24

CDS alerts can contribute to alert fatigue, which can lead to end-users ignoring evidence-based guidance. 9 Despite this, nudges, as described in the behavioral economics literature, which alter the environment in which decisions are made, can be an effective tool to guide users toward a new workflow. 25 26 Users in our study noted that because the alerts presented were unfamiliar and new, they would pause to take time to read the alert. Users also noted that, given their current workflows, they would encounter the new alerts created for this project infrequently. Participants stated that because the alerts were designed to pop up judiciously, they thought that the alerts would help guide them toward the evidence-based practice without becoming numb to the alert. Our study was unique in that it leveraged language and clicking actions thoughtfully to not point out that a provider was wrong, but to affirm the expertise of the user and gently nudge them into an alternative action.

Though usability testing facilitates effective implementation of CDS tools, resource constraints, including time, budgetary limitations, and staffing, can be barriers to incorporating usability testing into practice. 19 27 Our unique application of the rainbow spreadsheet allowed our team to rapidly incorporate user feedback and efficiently implement edits to the EHR tools. 16 This efficient, structured approach to eliciting feedback on the alerts enabled meaningful stakeholder engagement while maintaining the project's preimplementation timeline.

Our mixed-methods approach also allowed us to obtain qualitative and quantitative data around usability, while utilizing a brief survey to obtain additional information around users' attitudes.

Incorporating usability testing into our larger IS project allowed us to engage stakeholders, reflect on feasibility, and test our tools prior to deployment. 11 Utilizing these strategies aligns with previously published guidance around leveraging IS frameworks to customize CDS tools to improve implementation success. 11 28 Consistent with prior studies, our results demonstrated that time constraints were a significant barrier to utilizing new CDS tools. Our thoughtfully designed, easy-to-learn CDS tools should positively affect adoption and implementation. As part of the larger implementation project, we will provide routine feedback to clinicians, engage with departments to share updates, and offer technical support to promote uptake and effective use of the CDS tools. Ongoing clinician feedback will also inform continuous refinement of the alerts to strengthen usability, adoption, and implementation effectiveness.

This study has several limitations. First, this study was a single-site study with a small sample size. However, while the small sample size limits our ability to conclude from our quantitative survey data, as it was not powered to assess statistically significant results, we did reach thematic saturation, suggesting additional usability testing may not have provided additional benefits. Participants were self-selected, which may have introduced selection bias by overrepresenting clinicians with more favorable attitudes toward the EHR. In addition, usability testing was conducted in two academic outpatient practices, whereas the broader implementation project spans 19 additional sites, including non-academic practices and emergency departments. Accordingly, the study sample may not fully reflect the range of clinical settings and end users who will encounter these CDS tools in practice. Qualitative feedback may also have been influenced by social desirability bias, with participants potentially expressing more favorable views of the alerts during moderated sessions than they might in routine practice. 29 Finally, usability testing was conducted in the testing environment and not in live clinical settings, which may affect the generalizability of our results.

These limitations can affect the generalizability of our results. To address this issue, we will monitor the adoption and implementation of the CDS tools throughout our larger IS project to identify and implement needed changes. Future studies should include larger numbers of participants and a wider range of clinical areas to ensure depth of representation and minimize potential changes once fully implemented. Longitudinal studies should also be conducted to examine the effect of usability testing on clinical effectiveness measures.

## Conclusion

Though CDS tools have been demonstrated to serve as an effective strategy to improve the implementation of evidence-based guidelines into practice, time and budgetary restrictions can limit the ability of practices to incorporate user feedback into the design of tools. 27 Our study demonstrates an efficient, cost-effective way of incorporating stakeholder feedback into the development of CDS tools. Usability testing provided us with insights that allowed us to design alerts that aligned with existing clinical workflows, thus increasing the likelihood of success. 11 CDS stewardship guidelines highlight best practices to monitor and improve alerts. 10 Best practices include decreasing excessive firing, optimizing alert effectiveness, and establishing institutional governance to examine the overall burden of interruptive alerts. 10 Incorporating usability testing into larger, health system-wide IS initiatives can be an efficient, high-value strategy for strengthening stakeholder engagement and promoting adoption of evidence-based practices in routine clinical care. Beyond usability testing, we intend to communicate the value of CDS systems to providers and support adaptability to address technical challenges to improve functionalities during our meetings with practice sites. 11

## Notes

**Clinical Relevance Statement**
Incorporating usability-tested CDS alerts into clinical workflows can support more consistent, evidence-based antibiotic prescribing for pediatric ARTIs by reducing cognitive load and streamlining key tasks. Designing alerts that assume sound clinical judgment, while highlighting potential documentation gaps and offering embedded, actionable steps, may improve provider acceptance, reduce alert fatigue, and strengthen trust in the system. Routine engagement of clinicians in iterative alert refinement can enhance adoption, improve workflow alignment, and ultimately contribute to better AMS outcomes. Within a broader IS project, usability testing can strengthen intervention uptake, improve stakeholder engagement, and inform ongoing refinement during implementation.

## Notes

**Multiple-Choice Questions**

1. Which of the following was identified as a primary benefit of iterative usability testing for CDS alerts in this project?

- It eliminated the need for institutional governance approval.
- It reduced cognitive load and improved workflow integration.
- It ensured that all providers would use the alerts frequently.
- It replaced the need for evidence-based guideline references.

**Correct Answer** : The correct answer is option b. It reduced cognitive load and improved workflow integration. The manuscript reports that iterative usability testing substantially improved clarity, reduced cognitive load, and enhanced workflow alignment of the interruptive alerts. This was a major theme across waves, leading to improvements in satisfaction and adoption.

1. Which feature most improved clinicians' trust in the CDS alerts during usability testing?

- Adding more interruptive elements to ensure attention.
- Including citations to evidence-based guidelines within the alert.
- Making alerts longer and more descriptive.
- Requiring mandatory training prior to alert use.

**Correct Answer** : The correct answer is option b. Including citations to evidence-based guidelines within the alert. Participants indicated that hyperlinked citations to evidence-based guidance improved their trust in the narrow-spectrum antibiotic alert and reduced the need to seek external confirmation.
