# Supplementary material for: Optimizing Clinical Decision Support for Antibiotic Prescribing in Pediatric Acute Respiratory Tract Infections: A Usability Study
Source: Appl Clin Inform. 2026 Jul 22;17(3):615–24. doi: 10.1055/a-2906-3186 (PMC13391217; doi:10.1055/a-2906-3186)
Supplement: Supplementary file 1 — Supplementary Material [file 10-1055-a-2906-3186_29188937.pdf]

Confidential

Provider Usability Testing Survey

We appreciate you participating in our usability testing session. Please complete the survey below. All results are confidential.

How comfortable are you learning new technology at your job without guidance?	◯ Very uncomfortable<br>◯ Somewhat uncomfortable<br>◯ Undecided<br>◯ Somewhat comfortable<br>◯ Very comfortable
What resources have you used to learn about new Epic (EHR) functionality?	☐ Trial and error<br>☐ Tip sheets<br>☐ Online tutorials<br>☐ Peer assistance<br>☐ Epic's UserWeb<br>☐ TUHS IT's EPOCH Team<br>☐ Nothing<br>☐ Other
If you selected "Other," please provide further details:	____________________________________
What factors hinder your use of new technology?	☐ Lack of training<br>☐ Fear of making mistakes<br>☐ Time constraints<br>☐ Complexity of tools<br>☐ Lack of interest<br>☐ None of the above<br>☐ Other
If you selected "Other," please provide further details:	____________________________________
I feel like I have a bigger part in system-level change when participating in the development of new EHR tools.	◯ Strongly disagree<br>◯ Disagree<br>◯ Neither<br>◯ Agree<br>◯ Strongly Agree
I will share my experience in this process (i.e., usability testing) with my peers.	◯ Strongly disagree<br>◯ Disagree<br>◯ Neither<br>◯ Agree<br>◯ Strongly Agree
How many years have you worked at Temple? (Please round to the nearest whole number)	______________________________
How many years has it been since you completed your final training program or terminal degree? (Please round to the nearest whole number)	______________________________
Which clinical department do you practice in?	______________________________

Appl Clin Inform | DOI 10.1055/a-2906-3186 | © The Author(s).

What is your job title?	◯ Physician (MD/DO)<br>◯ Advanced Practice Provider (NP/PA)<br>◯ Resident Physician (MD/DO)<br>◯ Other
If "Other", please provide additional details	__________________________
Which age category do you fall into?	◯ < 25 years old<br>◯ 25-30 years old<br>◯ 31-35 years old<br>◯ 36-40 years old<br>◯ 41-50 years old<br>◯ 51-60 years old<br>◯ 61-70 years old<br>◯ >70 years old
Which of the following best describes your gender?	☐ Man<br>☐ Non-binary<br>☐ Woman<br>☐ Prefer to self-describe<br>☐ Prefer not to answer
Please provide further details around how you identify.	__________________________
Which of your following best describes your ethnicity?	☐ Hispanic<br>☐ Not Hispanic<br>☐ Other
If "Other," please provide additional details.	__________________________
Which of the following best describes your race? (Select all that apply)	☐ White<br>☐ Black or African American<br>☐ Asian<br>☐ Alaskan Native<br>☐ Native Hawaiian or Pacific Islander<br>☐ Other
If "Other", please provide additional details.	__________________________

Appl Clin Inform | DOI 10.1055/a-2906-3186 | © The Author(s).
